# Genome-wide association analyses of the 15^th ^QTL-MAS workshop data using mixed model based single locus regression analysis

**DOI:** 10.1186/1753-6561-6-S2-S5

**Published:** 2012-05-21

**Authors:** Wei-Xuan Fu, Chong-Long Wang, Xiang-Dong Ding, Zhe Zhang, Pei-Pei Ma, Zi-Qing Weng, Jian-Feng Liu, Qin Zhang

**Affiliations:** 1 Key Laboratory of Animal Genetics and Breeding of the Ministry of Agriculture, College of Animal Science and Technology, China Agricultural University, Beijing, 100193, China; 2 Institute of Animal Husbandry and Veterinary Medicine, Anhui Academy of Agricultural Sciences, Hefei 230031, China

## Abstract

**Background:**

The mixed model based single locus regression analysis (MMRA) method was used to analyse the common simulated dataset of the 15th QTL-MAS workshop to detect potential significant association between single nucleotide polymorphisms (SNPs) and the simulated trait. A Wald chi-squared statistic with *df *=1 was employed as test statistic and the permutation test was performed. For adjusting multiple testing, phenotypic observations were permutated 10,000 times against the genotype and pedigree data to obtain the threshold for declaring genome-wide significant SNPs. Linkage disequilibrium (LD) in term of *D' *between significant SNPs was quantified and LD blocks were defined to indicate quantitative trait loci (QTL) regions.

**Results:**

The estimated heritability of the simulated trait is approximately 0.30. 82 genome-wide significant SNPs (P < 0.05) on chromosomes 1, 2 and 3 were detected. Through the LD blocks of the significant SNPs, we confirmed 5 and 1 QTL regions on chromosomes 1 and 3, respectively. No block was detected on chromosome 2, and no significant SNP was detected on chromosomes 4 and 5.

**Conclusion:**

MMRA is a suitable method for detecting additive QTL and a fast method with feasibility of performing permutation test. Using LD blocks can effectively detect QTL regions.

## Background

Recently, the high-density single nucleotide polymorphism (SNP) arrays have been developed for almost all domestic animals, which offer the prerequisite of genome-wide association study (GWAS), a more powerful approach for high-resolution mapping of loci controlling phenotypic traits in domestic animals [1]. In GWAS, two basic designs of resource population have been widely used for association analysis, one is the case-control design with unrelated individuals, and the other is the family-based design with pedigree structure. Corresponding to these two designs, different approaches for association analysis have been proposed. However, there is no clear evidence showing general superiority of one approach over others. In farm animals, family based design is more relevant because of complex pedigree structure in almost all animal populations. In our previous GWAS study [2], we employed a mixed model based single locus regression analysis (MMRA) to test the association between SNPs and milk production traits in dairy cattle. We found this method was more powerful than the TDT-based single locus regression analysis. To further verify its performance in terms of power and type I error, we applied it to the common dataset provided in the 15th QTL-MAS workshop.

## Methods

The simulated population consisted of 3,220 individuals in two generations. The first generation consisted of 20 sires and 200 dams, which were assumed to be unrelated. Each sire mated with 10 dams and each dam produced 15 progenies, leading to a total of 3,000 individuals in the second generation. Of the 15 progenies of each dam, 10 were phenotyped for a continuous trait. All of the 3,220 individuals were genotyped for 9,990 SNP markers distributed on 5 chromosomes without missing. Each chromosome had a size of 1 Morgan (M) and carried 1,998 evenly distributed SNPs.

### Variance component estimation

We applied the software DMU (Version 6, release 5.0) [3] to estimate the variance components of the simulated trait, which would be used in the subsequent association analysis, based on the following model

y=1μ+Za+e

Where **y **is the vector of phenotypes of the 2,000 phenotyped individuals, *μ *is the overall mean, **a **is the vector of the residual polygenic effect with $a\sim N\left(0,A\sigma_{a}^{2}\right)$ (where **A **is the additive genetic relationship matrix and $\sigma_{a}^{2}$ is the additive genetic variance), Z is the incidence matrix of a, and e is the vector of residual errors with $e\sim N\left(0,I\sigma_{e}^{2}\right)$ (where **I **is a unit matrix and $\sigma_{e}^{2}$ is the residual error variance).

### Genotype quality control

We removed the 1,000 progenies without phenotypes off the genotype data, and we calculated the minor allele frequency (MAF) for each SNP for the remained 2,220 individuals (2,000 progenies and 220 parents). We found that 2,879 SNPs were homozygous (MAF = 0) for all the tested individuals and additionally 715 SNPs had a MAF less than 0.03. These SNPs were removed and 6,396 SNPs remained for the subsequent analyses.

### Association analysis

The mixed model based single locus analysis [2,4] was performed based on the following linear mixed model:

y=1μ+bx+Za+e

where **y **is the vector of phenotypes of the 2000 phenotyped individuals, *μ *is the overall mean, × is the vector of the SNP genotype indicators which takes values 0, 1 or 2 corresponding to the three genotypes 11, 12 and 22 (assuming 2 is the allele with a minor frequency), *b *is the regression coefficient of phenotypes on SNP genotypes (i.e., the substitution effect of the SNP), **a **is the vector of the residual polygenic effect with $a\sim N\left(0,A\sigma_{a}^{2}\right)$, **Z **is the incidence matrix of **a**, and **e **is the vector of residual errors with $e\sim N\left(0,I\sigma_{e}^{2}\right)$.

For each SNP, the estimate of b and the corresponding sampling variances $Var\left(\overset{\wedge }{b}\right)$ can be obtained via mixed model equations (MME), and a Wald chi-squared statistic ${\hat{b}}^{2}/Var\left(\hat{b}\right)$ with *df *=1 was constructed to examine whether the SNP is associated with the trait.

### Statistical inference

For the analyses above, the permutation method was adopted to adjust for multiple testing from the number of SNP loci detected. In our method, the phenotypes were permuted 10,000 times against the genotype and pedigree data and the empirical distribution of the Wald chi-squared statistic under the null hypothesis (no association existed between any SNP and the trait in genome-wide level) was obtained using the largest Wald chi-squared statistic value across all SNPs from each permuted dataset. The threshold value for declaring a significant association was determined by choosing the 95th percentile of the empirical distribution, i.e., we declared a significant SNP at a 0.05 genome-wide significance level if its raw value of the Wald chi-squared statistic was larger than the empirical threshold value.

For the significant SNPs, linkage disequilibrium (LD) in term of *D' *between them was quantified using Haploview [5] and the LD blocks were defined by the criteria of Gabriel et al. [6] with default parameters.

## Results and discussion

### Association analysis results

**Figure 1:**
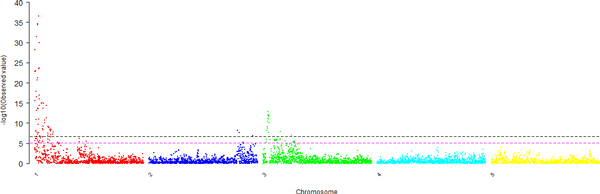
**The Manhattan plots of GWAS for the 15th QTL-MAS Workshop Data**. Chromosomes 1-5 are shown with different colours. The magenta horizontal dotted line indicates the significance threshold of Bonferroni correction (-log10(7.82E-6)), and the black one indicates that of permutation test(-log10(2.31E-7)).

The estimates of $\sigma_{a}^{2}$ and $\sigma_{e}^{2}$ are 24.82 and 58.65, respectively, so that the heritability estimate is 0.30 approximately. The profile of the raw p values (from the chi-distribution and in terms of -log10 p) of all tested SNPs is shown in Figure 1. By using simply Bonferroni correction, we detected 119 significant SNPs of 0.05 genome-wide significance level (raw *p *values < 7.82E-6). However, by using permutation test, we detected 82 significant SNPs of 0.05 genome-wide significance level (raw *p *values < 2.31E-7) for the simulated trait. The 82 significant SNPs are distributed on chromosomes 1, 2 and 3, *i.e*., 63 on chromosome 1, 3 on chromosome 2, and 16 on chromosome 3. The significant SNPs on chromosome 1 cover a large interval between 0.15cM and 15.30cM. The simulated SNP with the biggest effect is at 2.90cM (No.58), while the most significant SNP identified is at 3.55cM (No.71). The positions of the 3 significant SNPs on chromosome 2 are 81.90cM, 83.10cM and 95.80cM, respectively. Moreover, 13 of the 16 significant SNPs on chromosome 3 cover an interval between 4.25cM and 5.65cM, and the other 3 are at 3.70cM, 16.10cM and 26.75cM, respectively. No SNPs on chromosome 4 and 5 were found to be associated with the trait significantly. If we set the significant level at 0.01 for the permutation test, the number of significant SNPs reduces to 32, of which 3 are on chromosome 3 and all others on chromosome 1.

**Figure 2:**
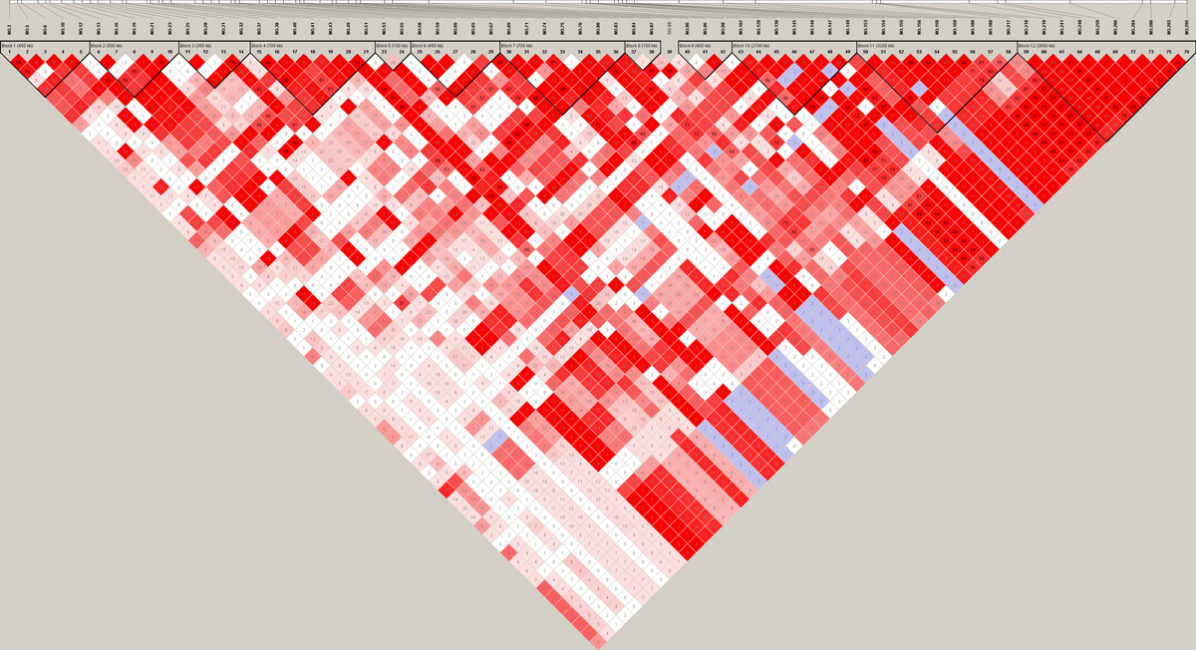
**Linkage disequilibrium (LD) patterns for significant SNPs on chromosome 1 (a), 2 (b) and 3 (c)**. Values in boxes are D' values between SNP pairs and the boxes are coloured according to the standard Haploview colour scheme: LOD>2 and D'=1, red; LOD>2 and D'<1, shades of pink/red; LOD<2 and D'=1, blue; LOD<2 and D'<1, white (LOD is the log of the likelihood odds ratio, a measure of confidence in the value of D'). LD blocks are marked with triangles.

**Figure 3:**
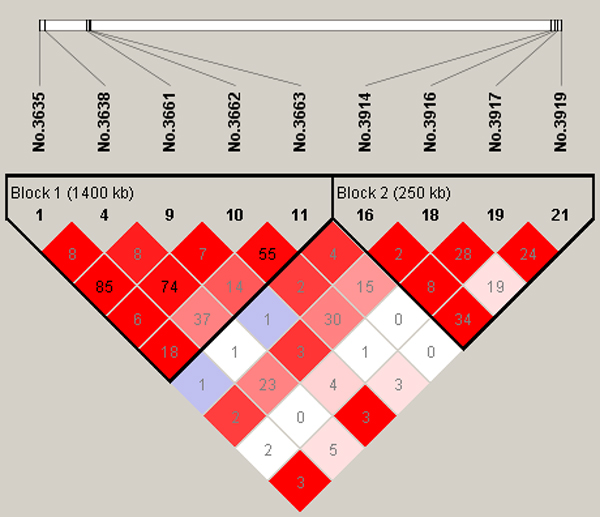
**Linkage disequilibrium (LD) patterns for significant SNPs on chromosome 2**. The true simulated QTN (No.3875 and No.4300, respectively) are also included in addition to the significant SNPs.

**Figure 4:**
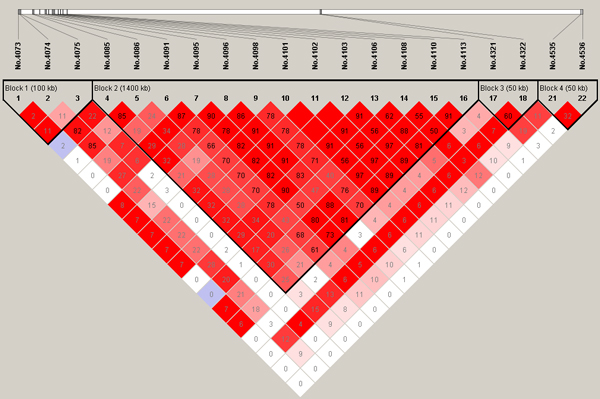
**Linkage disequilibrium (LD) patterns for significant SNPs on chromosome 3**. The true simulated QTN (No.3875 and No.4300, respectively) are also included in addition to the significant SNPs.

To further pinpoint the relationship among the detected SNPs, we analysed the LD levels in terms of D' between the significant SNPs (Figures 2, 3 and 4) for chromosomes 1-3, respectively. Through the criteria of Gabriel et al. [6] with default parameters in Haploview [5], we defined 5 LD blocks on chromosome 1, which harbour 4 to 10 significant SNPs, and 1 LD block on chromosome 3, which harbour 10 significant SNPs with 6 outside. No block was detected on chromosome 2. The LD patterns show that these significant SNPs links to each other in different LD levels.

### Comparison of the significant SNPs with the simulated QTN

On chromosome 1, there is one simulated QTN located at 2.85cM (No.57), which had the largest effect among all simulated QTNs. We detected 63 significant SNPs on this chromosome. However, the true QTN at 2.85cM has a MAF of 0 and was discarded after quality control, and the adjacent SNP at 2.90cM (No.58), which has the largest estimated effect among all significant SNPs, is accordingly considered as the putative QTN. Although a large number of pseudo significant SNPs were identified on this chromosome, the LD levels between the most effective SNP and other 62 significant SNPs (Figure 2) showed that 47 of them are in strong LD (*D'*>0.5) with it. This suggests that the simulated QTN may be surrogated by a suite of "ghost" QTNs nearby due to high LD level.

On chromosome 2, there are two simulated QTNs in coupling linkage phase located at 81.90cM (No.3638) and 93.75cM (No.3875), respectively. We detected 3 significant SNPs, the first is exactly at 81.90cM, and the second (No.3662, at 83.10cM) is in strong LD (*D' *= 0.97) with the first one (Figure 3). But the third one No.3916 is at 95.80cM and is 2.05cM away from the second simulated QTN, while the LD level between them is strong (*D'*=0.69, Figure 3).

On chromosome 3, there are two simulated QTN in repulsion linkage phase located at 5cM (No.4100) and 15cM (No.4300), respectively. However, the first simulated SNP on this chromosome also has a MAF of 0 and was discarded after quality control. Of the 16 significant SNPs detected, 10 are harboured in the LD block covering the interval between 4.75cM and 5.65cM with an average LD level of 0.97 (*D'*), in which SNP No.4101 is just adjacent to the first true QTN. The second simulated QTN is 1.10cM away from the significant SNP (No.4322) and the LD level between them is strong (*D'*=0.93).

The one simulated imprinting QTN on chromosome 4, and 2 simulated epistatic QTNs on chromosome 5 were not detected by our analysis. This is because our method dose not account for both imprinting effect and epistatic effect. Our method needs to be further improved to account for interaction effects between SNPs and imprinting effects from parents.

### Comparison of the significant SNPs with the those with high effects estimated via Bayesian approaches

To further validate significant SNPs identified herein, we compared the most promising SNPs detected with those with highest effects estimated via Bayesian approaches (BayesA, BayesB and BayesCπ) reported in our another analysis on prediction of genomic breeding values for the same data set [7]. Since the results from the three Bayesian approaches are similar and BayesCπ performed best, we only compare with BayesCπ here. Specifically, on chromosome 1, the most effective SNP (No.58) identified by MMRA is exactly the same as that by BayesCπ. On chromosome 2, BayesCπ revealed SNP No. 3660 with the largest effect and SNP No.3873 with the second largest effect, which are close to and in strong LD with the significant SNPs No. 3662 and No. 3916 detected by MMRA. On chromosome 3, the two promising SNPs detected by MMRA are No.4101 and No.4322, which are close to the SNP with the largest (No.4092) and the second largest (No.4331) effect estimated by BayesCπ, respectively. In all, most of findings herein are largely consistent with those with highest effect estimates via BayesCπ. This further demonstrates that the Bayesian approaches (particularly BayesCπ) could also sever as tools for QTL mapping, as suggested by Fan et al. [8].

### Computing time

All analyses were implemented through Fortran programs and performed on an octal-core Linux Server (Intel Xeon E5504 2.00GHz; 48.00GB RAM). The time needed was about 1.5 minutes for one permutation analysis. The 10,000 permutations were performed through 8 threads, each was assigned 1,250 permutations. So, the total computing time was about 31 hours. This shows that MMRA is a fast method with feasibility of performing a large number of permutations.

## Conclusion

Our results herein show that the MMRA method is suitable for detecting additive QTL, and it is a fast method with feasibility of performing permutation test. And the LD region on chromosome 3 can effectively integrate significant SNPs for QTL region detection. However, we detects only one true additive QTN (No.3638), two SNPs (No.58 and No.4101) close to two true additive QTNs (No.57 and No.4100) with many false positives, which remains to be further investigated and the MMRA method needs to be further improved to account for other non-additive effects.

## List of abbreviation used

GWAS: Genome-Wide Association Study; MMRA: Mixed Model based Single Locus Regression Analysis; SNP: Single Nucleotide Polymorphisms; QTL: Quantitative Trait Locus; QTN: Quantitative Trait Nucleotide; LD: Linkage disequilibrium; M: Morgan; MAF: Minor Allele Frequency; TDT: Transmission Disequilibrium Test.

## Competing interests

The authors declare that they have no competing interests.

## Authors' contributions

WXF, CLW, XDD, ZZ, PPM and ZQW carried out the analyses and contributed the manuscript. JFL and QZ coordinated the analyses and drafted the write-up. All authors have read and contributed to the final text of the manuscript.
